# Germ Cell Tumor With Somatic-Type Malignancy: A Case Report and Review of the Literature

**DOI:** 10.7759/cureus.25879

**Published:** 2022-06-12

**Authors:** Ahmad Cheema, Fakeha Siddiqui, Amir Kamran

**Affiliations:** 1 Hematology-Oncology, West Virginia University, Morgantown, USA; 2 Internal Medicine, Camden Clark Medical Center, Parkersburg, USA

**Keywords:** retroperitoneal mass, adenocarcinoma, malignant transformation, somatic-type malignancy, non-seminoma germ cell tumor

## Abstract

A malignant germ cell tumor (GCT) might contain or transform into malignant non-germ cell histology, commonly referred to as somatic-type malignancy (SM). It is a rare phenomenon with poorly understood pathogenesis. SMs are mostly associated with teratomas and are mainly observed in late relapsing cases. There are no consensus guidelines on the management of SMs; however, surgery is considered to be the mainstay of treatment. Prognosis is variable depending on the time of diagnosis, site of relapse, and type of histology.

Here, we present a case of a 44-year-old male with a history of mixed GCT stage IIA, initially managed with right radical orchiectomy, who developed a relapse of GCT 10 years later with an SM of adenocarcinoma subtype.

## Introduction

In the year 2022, there will be approximately 9910 estimated new individuals diagnosed with testicular cancer in the United States, representing approximately 0.5% of all cancer cases [1]. Even though testicular cancer is uncommon, it is the most common malignancy in men aged 20-39 years [2]. Most testicular cancers are germ cell tumors (GCTs), classified as seminomatous and non-seminomatous germ cell tumors (NSGCTs). Of NSGCTs, 30-50% are comprised of more than one germ cell component and hence are called mixed GCTs [3]. It is a highly curable cancer with a five-year relative survival rate of 80-95% when treated with a multimodal approach [1,4,5]. Despite the good prognosis of testicular GCTs, 10-30% of patients relapse after initial treatment with some experiencing a late relapse, which is defined as recurrence of a GCT at least two years after initial complete remission [6]. In a small number of patients, especially those experiencing a late relapse, malignant GCTs might contain or transform into malignant non-germ cell histologies resembling cancers seen in other organs, which are known as somatic-type malignancies (SMs) [7].

Here, we report a case of mixed GCT presenting a late relapse with an SM of adenocarcinoma type.

## Case presentation

A 44-year-old male with a history of right non-seminomatous testicular cancer diagnosed 10 years ago presented with abdominal discomfort. At the time of initial diagnosis of testicular cancer, the patient underwent right radical orchiectomy and retroperitoneal lymph node dissection. Pathology was consistent with mixed GCT: teratoma (50%), embryonal carcinoma (30%), and yolk sac tumor (15%). Surgical margins were negative. Four out of eight lymph nodes were positive for involvement with predominantly embryonal carcinoma subtype. The final stage was stage IIA (pT2 pN1 M0). In the adjuvant setting, the patient was started on etoposide and cisplatin; however, he decided not to continue chemotherapy after one cycle due to poor tolerance. Follow-up imaging showed no evidence of disease. Four years later, the patient underwent computed tomography (CT) scan of the abdomen for abdominal pain, which revealed a single retroperitoneal lymph node to the left of the aorta measuring 6 x 8 mm. Unfortunately, the patient did not follow up with medical oncology for several years after this scan.

**Figure 1:**
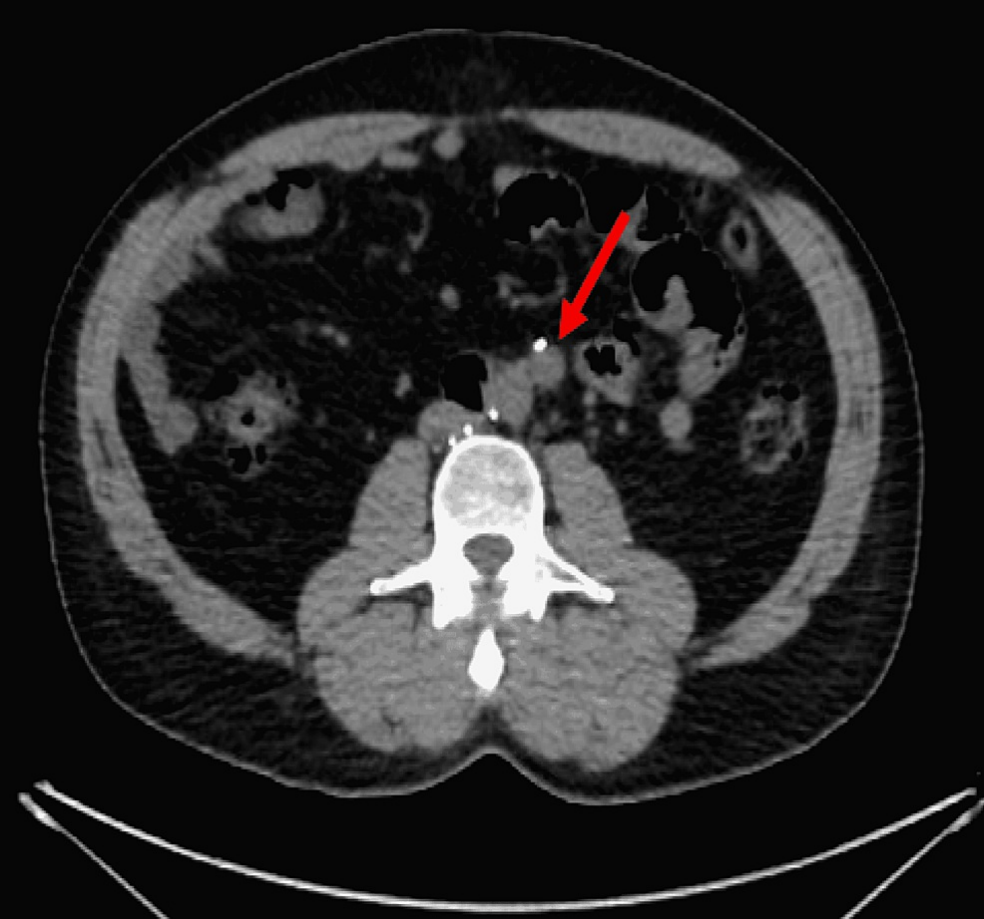
Computed tomography of the abdomen showing an enlarged retroperitoneal lymph node.

**Figure 2:**
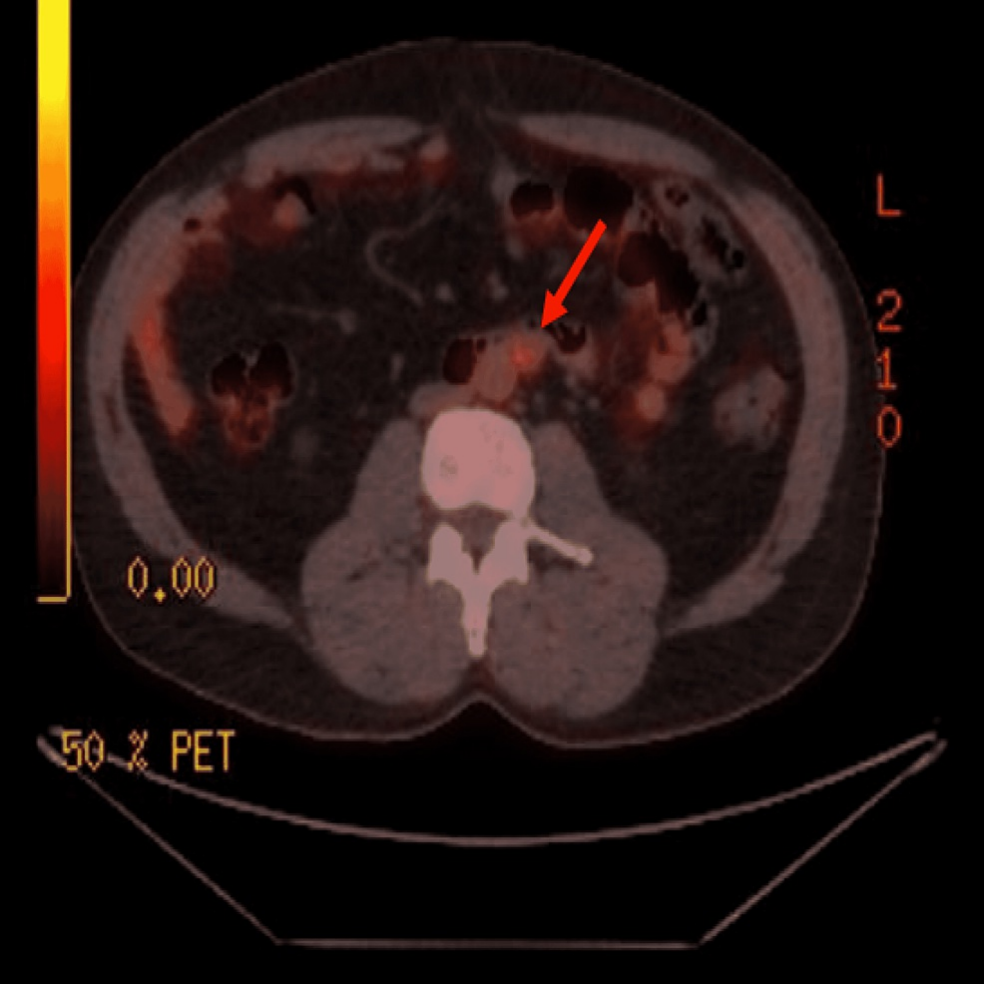
Positron emission tomography scan showing an enlarged retroperitoneal lymph node with maximum standard uptake value of 3.

The patient was in his usual state of health until two months ago when he started to experience intermittent mid-abdominal discomfort with no identifiable worsening or alleviating factors. He did not report fever, nausea, vomiting, abdominal cramping, diarrhea, or constipation. He denied weight loss, palpable lymph nodes, or testicular mass. Given the patient’s prior history of testicular cancer with involvement of retroperitoneal lymph nodes, a contrast-enhanced CT of the abdomen was performed, which showed an increase in the size of the retroperitoneal lymph node previously seen six years ago. This was followed by a positron emission tomography (PET) scan, which showed an increase in the size of the single enlarged lymph node in the retroperitoneum, now measuring 13 x 13 mm compared to 6 x 6 mm previously (Figure 1). This lymph node had a maximum standard uptake value (SUVmax) of 3 (Figure 2).

No other areas of enlarged or hypermetabolic lymphadenopathy were noted. While serum lactate dehydrogenase (LDH) was mildly elevated at 286 U/L, serum alpha-fetoprotein (AFP) was normal and serum beta-human chorionic gonadotropin was undetectable.

The patient underwent surgical excision of the retroperitoneal lymph node. Tumor cells were positive for CK20 and negative for CK7, consistent with metastatic adenocarcinoma of likely colon as the primary site of origin. This was followed by a colonoscopy, but no mass was noted in the entire colon. Given the unusual site of metastasis from colon cancer, absence of a colonic mass, and prior history of retroperitoneal lymph node metastasis from testicular cancer, further workup was directed toward evaluation for a relapse of testicular cancer. Tumor cells showed weak SALL4 staining. Tissue slides from initial orchiectomy and retroperitoneal lymph node dissections were obtained and reviewed again, and it was noted that the original tumor contained primarily immature teratoma showing differentiation as immature neuroectoderm, sarcomatous stroma, odontogenic epithelium, squamous epithelium, and adenocarcinoma. The adenocarcinoma component was morphologically and immunophenotypically similar to the one seen in the most recent retroperitoneal lymph node specimen, suggesting metastasis from a relapsed GCT with transformation into an SM of adenocarcinoma subtype.

## Discussion

SM arising from a GCT is a rare phenomenon, occurring in approximately 2.7% to 8.6% of GCT cases, and is more commonly observed in late relapse cases [8-10]. SMs have mostly been described with teratomas; however, non-teratomatous associations have been observed as well [11]. SMs can develop in the primary testicular tumor as well as in metastatic sites, and encompass a wide variety of histologic subtypes with sarcomas being the most common followed by carcinomas and primitive neuroectodermal tumors (PNET) [7,9,12]. Rarely, SMs can be hematological malignancies, a combination of different forms, or undifferentiated tumors [7,13]. Hwang et al., in a study of 63 cases, observed that rhabdomyosarcoma was the most common histological subtype of SM for tumors found in the testis, while carcinoma, particularly adenocarcinoma, was the predominant subtype found in the metastatic sites, as in our case [14].

Given the rarity of SMs, the pathogenesis is poorly understood. Several theories have been postulated in the past. One hypothesis is that SM may result from the transformation of a teratomatous component in the GCT, as Hwang et al. observed that a teratomatous component was present in 91% of GCTs in the testis and 87% of GCTs in metastatic sites [14]. Another hypothesis is that the SMs and corresponding GCTs are clonally related and likely derived from a common pluripotent progenitor cell. This was studied by Kum et al. involving 27 pairs of teratoma and SMs in metastatic lesions, and showed that SMs that developed in GCTs had the same genetic alterations as in the corresponding teratomas, detectable by fluorescence in situ hybridization (FISH) and loss of heterozygosity studies [15]. Similarly, Umbreit et al. demonstrated high concordance between teratomas and adjacent somatic transformation based on targeted DNA and RNA sequencing [16]. Finally, the finding of SMs within the metastatic lesions in late relapses with a history of chemotherapy for the original GCT has prompted a hypothesis that SMs may develop from chemoresistant elements of the original GCT. It is believed that chemotherapy mainly affects the more aggressive components of the tumor, while the less aggressive components may later undergo genetic changes and acquire a more malignant potential. However, this was not observed to be true in a study by Magers et al. where 13 patients developed SMs before receiving any chemotherapy [11].

As SMs are mostly seen at metastatic sites in late relapses, even decades after initial diagnosis of GCT, it may be difficult to recognize the origin of SM from a GCT, especially given the fact that not all SMs would have a concomitant GCT. History of GCT may be helpful in testing additional immunohistochemical stains like SALL4, which is an excellent marker for GCT; however, a negative or weakly positive SALL4 staining would not rule out a GCT origin. In our case, metastatic adenocarcinoma of colonic origin was diagnosed initially with weakly positive staining for SALL4; however, the morphological and immunophenotypic similarity to the adenomatous component of the original mixed GCT suggested relapsed GCT with SM of adenocarcinoma type.

At present, the optimal strategy for the management of SMs is unclear. Several studies have shown resistance of SMs to GCT-oriented chemotherapy with the exception of one report from Pantaleo et al. of successful treatment of a mixed GCT with SM of sarcoma type with a GCT-oriented treatment [17-19]. A study of 121 patients with SM treated at Indiana University demonstrated a clinical complete remission (CR) rate of 12.8% with cisplatin-based chemotherapy, which is significantly lower than the 70-80% rate of CR that would be expected with cisplatin-based chemotherapy in patients with metastatic GCT [18]. It is unclear whether chemotherapy directed toward the transformed component would help achieve better results or not. The role of radiation in this setting is not well defined either. Therefore, surgical resection of the primary or metastatic site, when resectable, is currently believed to be the mainstay of treatment for SMs to prolong survival [10,18].

In terms of prognosis of GCTs with SMs, Sharma et al. observed that patients who had GCT with SM at first presentation or initial diagnosis had a five-year overall survival (OS) rate of 87.5% compared to those who presented with SM in relapse or post-chemotherapy when five-year OS rate dropped to 37-40% [20]. Several other studies have suggested that the histological subtype of SM may affect the prognosis as well, with carcinomatous histology conferring a worse prognosis than sarcomatous or primitive PNET histologies [8,14,18]. Moreover, Hwang et al. observed that patients with metastatic SMs had significantly poorer clinical outcomes compared to patients with SMs limited to the testes [14].

## Conclusions

SMs arising from GCTs are rare with a poorly understood pathogenesis. These are usually observed in late relapsing cases mainly at metastatic sites. Surgical resection remains the mainstay of treatment since the chemoresistance of SMs has been demonstrated in clinical studies. Prognosis is worse for late relapsing cases, SM in metastatic lesions, and carcinomatous histology.

## References

[1] (2022). Cancer stat facts: testicular cancer. https://seer.cancer.gov/statfacts/html/testis.html.

[2] Miller KD, Fidler-Benaoudia M, Keegan TH, Hipp HS, Jemal A, Siegel RL (2020). Cancer statistics for adolescents and young adults, 2020. CA Cancer J Clin.

[3] Cheville JC (1999). Classification and pathology of testicular germ cell and sex cord-stromal tumors. Urol Clin North Am.

[4] Carver BS, Serio AM, Bajorin D (2007). Improved clinical outcome in recent years for men with metastatic nonseminomatous germ cell tumors. J Clin Oncol.

[5] Mostofi FK (1973). Proceedings: testicular tumors. Epidemiologic, etiologic, and pathologic features. Cancer.

[6] Baniel J, Foster RS, Gonin R, Messemer JE, Donohue JP, Einhorn LH (1995). Late relapse of testicular cancer. J Clin Oncol.

[7] Mikuz G, Colecchia M (2012). Teratoma with somatic-type malignant components of the testis. A review and an update. Virchows Arch.

[8] Comiter CV, Kibel AS, Richie JP, Nucci MR, Renshaw AA (1998). Prognostic features of teratomas with malignant transformation: a clinicopathological study of 21 cases. J Urol.

[9] Motzer RJ, Amsterdam A, Prieto V (1998). Teratoma with malignant transformation: diverse malignant histologies arising in men with germ cell tumors. J Urol.

[10] Washino S, Konishi T, Saito K, Ohshima M, Nakamura Y, Miyagawa T (2017). Two cases of somatic-type malignancy as a very late relapse of testicular cancer successfully managed by surgical resection. J Surg Case Rep.

[11] Magers MJ, Kao CS, Cole CD, Rice KR, Foster RS, Einhorn LH, Ulbright TM (2014). "Somatic-type" malignancies arising from testicular germ cell tumors: a clinicopathologic study of 124 cases with emphasis on glandular tumors supporting frequent yolk sac tumor origin. Am J Surg Pathol.

[12] Necchi A, Colecchia M, Nicolai N (2011). Towards the definition of the best management and prognostic factors of teratoma with malignant transformation: a single-institution case series and new proposal. BJU Int.

[13] Zeh N, Wild PJ, Bode PK, Kristiansen G, Moch H, Sulser T, Hermanns T (2013). Retroperitoneal teratoma with somatic malignant transformation: a papillary renal cell carcinoma in a testicular germ cell tumour metastasis following platinum-based chemotherapy. BMC Urol.

[14] Hwang MJ, Hamza A, Zhang M, Tu SM, Pisters LL, Czerniak B, Guo CC (2022). Somatic-type malignancies in testicular germ cell tumors: a clinicopathologic study of 63 cases. Am J Surg Pathol.

[15] Kum JB, Ulbright TM, Williamson SR (2012). Molecular genetic evidence supporting the origin of somatic-type malignancy and teratoma from the same progenitor cell. Am J Surg Pathol.

[16] Umbreit EC, Siddiqui BA, Hwang MJ (2020). Origin of subsequent malignant neoplasms in patients with history of testicular germ cell tumor. Cancers (Basel).

[17] Guo CC, Punar M, Contreras AL, Tu SM, Pisters L, Tamboli P, Czerniak B (2009). Testicular germ cell tumors with sarcomatous components: an analysis of 33 cases. Am J Surg Pathol.

[18] Rice KR, Magers MJ, Beck SD, Cary KC, Einhorn LH, Ulbright TM, Foster RS (2014). Management of germ cell tumors with somatic type malignancy: pathological features, prognostic factors and survival outcomes. J Urol.

[19] Pantaleo MA, Mandruzzato M, Indio V (2021). Case report: the complete remission of a mixed germ cell tumor with somatic type malignancy of sarcoma type with a GCT-oriented therapy: clinical findings and genomic profiling. Front Oncol.

[20] Sharma A, Alifrangis C, Milic M (2019). Somatic transformation in metastatic testicular germ cell tumours - a different disease entity. Anticancer Res.

